# regCOVID: Tracking publications of registered COVID-19 studies

**DOI:** 10.1186/s12874-022-01703-9

**Published:** 2022-08-10

**Authors:** Craig S. Mayer, Vojtech Huser

**Affiliations:** Lister Hill National Center for Biomedical Communication, National Library of Medicine, NIH, Bethesda, MD USA

**Keywords:** COVID-19, Data Science, Clinical Trials, Result Publications, Informatics

## Abstract

**Background:**

In response to the COVID-19 pandemic many clinical studies have been initiated leading to the need for efficient ways to track and analyze study results. We expanded our previous project that tracked registered COVID-19 clinical studies to also track result articles generated from these studies. Our objective was to develop a data science approach to identify and analyze all publications linked to COVID-19 clinical studies and generate a prioritized list of publications for efficient understanding of the state of COVID-19 clinical research.

**Methods:**

We conducted searches of ClinicalTrials.gov and PubMed to identify articles linked to COVID-19 studies, and developed criteria based on the trial phase, intervention, location, and record recency to develop a prioritized list of result publications.

**Results:**

The performed searchers resulted in 1 022 articles linked to 565 interventional trials (17.8% of all 3 167 COVID-19 interventional trials as of 31 January 2022). 609 publications were identified via abstract-link in PubMed and 413 via registry-link in ClinicalTrials.gov, with 27 articles linked from both sources. Of the 565 trials publishing at least one article, 197 (34.9%) had multiple linked publications. An attention score was assigned to each publication to develop a prioritized list of all publications linked to COVID-19 trials and 83 publications were identified that are result articles from late phase (Phase 3) trials with at least one US site and multiple study record updates. For COVID-19 vaccine trials, 108 linked result articles for 64 trials (14.7% of 436 total COVID-19 vaccine trials) were found.

**Conclusions:**

Our method allows for the efficient identification of important COVID-19 articles that report results of registered clinical trials and are connected via a structured article-trial link. Our data science methodology also allows for consistent and as needed data updates and is generalizable to other conditions of interest.

## Introduction

The COVID-19 pandemic led to the initiation of thousands of clinical studies testing various interventions and studying the natural course of the disease [1, 2]. This included the study of a wide variety of both novel and repurposed interventions to prevent or treat COVID-19 [3]. For researchers or the public, it can be difficult to navigate and organize a large number of such studies. We previously created a framework for monitoring registered COVID-19 studies using ClinicalTrials.gov (CTG) registry, known as regCOVID [4, 5]. The framework uses data science methods that computationally identifies COVID-19 clinical studies using a keyword search. The framework also uses a computerized code to regularly monitor and analyze key features relating to COVID-19 interventional trials, observational studies, and patient registries registered at CTG.

A study may publish three types of information: [1] registration data at study initiation (in a clinical trial registry, such as CTG), [2] basic summary results at study completion (in a clinical trial registry), or [3] an article with well commented full study results (in a journal). Prior analyses of phase-2-or-higher interventional trials indicate that only 27.8% publish a study result article [6]. A completed study with one or more study results journal articles provides the most value to researchers and the public. Poor information about study status or study results may lead to reduced public trust in clinical trials enterprise [7].

In this study, we extended our regCOVID monitoring project to now identify study result articles that are linked to registered COVID-19 trials [4, 6]. Since the total amount of all published COVID-19 articles may be overwhelming [8, 9], we propose focusing only on articles that are linked to formally registered studies to facilitate an effective review of COVID-19 scientific literature. Unlike many efforts that use predominantly manual review to provide the public with an overview of trials and their results [10], we use a computational clinical research informatics approach to assess which COVID-19 studies are publishing, what they are publishing and when [11]. A reader may have limited time to read and review articles or abstracts and finding a way to prioritize articles is key in allowing for efficient review.

Therefore, the purpose of this research is to develop a data science driven methodology to identify published clinical trial results article and prioritize which articles clinical researchers, health care professionals and interested individuals can read to better understand the current state of clinical trial research for COVID-19. Our computerized processing script can also be generalized and applied to other conditions.

## Materials and methods

Our project repository (available at https://github.com/lhncbc/r-snippets-bmi/tree/master/regCOVID/regCOVIDpublications) includes our computer code, supplemental files, analysis results and a detailed web-based results report [12]. We also refer to the project using a short name of regCOVIDpub. Throughout the methods and results, we reference supplemental files on the project repository by the file name. The script is written in R language. For result reports, we use R Markdown framework. For most analyses, the repository will offer monthly refreshed results.

To find result articles linked to COVID-19 clinical studies registered on CTG, we perform three high-level steps. In the first step, COVID-19 studies are identified. In the second step we attempt to gather all published study result articles linked to those studies, and in the third step, we retrieve additional metadata about the articles and their affiliated studies and create a prioritization scoring system to identify the most significant publications. The sections below elaborate on details of each high-level step.

### COVID-19 studies

For the first step all CTG registered COVID-19 studies were retrieved (see supplemental file ‘../regCOVIDpublications_trials_all.csv’ in the study repository) using the results of our previously published work on tracking registered COVID-19 clinical studies (regCOVID) [4]. Eligible studies used for analysis included all COVID-19 interventional trials, observational studies or registries that were registered on CTG and were recruiting, active, or ended (completed or terminated). All studies regardless of country and site locations were included as long as they were registered on CTG.

### Identification of COVID-19 research articles

Once the eligible studies were identified, in the second step, we searched for publications linked to each study using two different methods: registry-linked and abstract-linked. This methodology is based on prior published work by our research group [6]. We describe each article linkage mechanism separately below.

### Registry-linked result article search

Registry linked result articles are those included in the study record on the CTG registry. We used the Aggregate Analysis of ClinicalTrials.gov (AACT) database developed by researchers at Duke University [13]. The AACT database is created by parsing the XML study data from CTG [13]. The ‘result_reference’ XML field within the study record was used to identify result publications for the study. Using prior knowledge that some result_reference articles are incorrectly labelled as such, we used article publication date to remove misclassified articles (that were actually of type ‘supporting_reference’). See this prior publication for details [6]. We then linked the results publications found in the CTG study records to the PubMed abstract to identify key details about the article, such as article title and type. For context, a prior study on a set of 8 907 trials completed between 2006 and 2009 found that 7.3% of trials tend to have at least one registry-linked result article [6].

### Abstract-linked result article search

Abstract linked articles are those where authors of trial result articles follow guidance of the International Committee of Medical Journal Editors and reference properly the relevant trial identifier in the article abstract. This reference is processed by PubMed and turned into searchable article metadata (called secondary identifier). We retrieved abstract linked articles by a metadata search in PubMed as articles where the article secondary identifier contained a CTG identifier (NCT ID) of a COVID-19 trial. For context, the same previously mentioned prior study found that 23.3% of trials tend to have abstract-linked result articles [6].

We combined the lists of publications from these two search methods to generate a master list of linked COVID-19 articles (see supplemental file ‘regCOVIDpublications_publication_list_all.csv’). The master publication list allows for an enhanced review of the resulting articles. It combines PubMed and CTG data and shows the trial NCT identifier, PubMed PMID identifier, trial intervention (e.g., convalescent plasma), article keywords using Medical Subject Headings (MeSH), trial sponsor (e.g., University of Oxford) and many other article or trial metadata. We separated the article set based on study type and performed the rest of the analysis on just interventional trials, as they are the most relevant trials (at this point in the pandemic) and the main focus of our study.

### Analysis of publications

#### Interventions

The intervention being studied (e.g., remdesivir) in a trial and discussed in a publication contributes to how significant the publication is in the research landscape. Interventions must progress through the phases of interventional trials (Phase 1/2/3) to receive regulatory approval for a given indication. Interventions gain significance in application as they progress through the different trial phases as they reach a point to apply for regulatory approval and widespread application with a successful Phase 3 trial. Many interventions may not reach Phase 3 trials and never reach a stage of regulatory approval. Different interventions were studied for COVID-19 and advanced to different phases. Therefore, we created an intervention significance score for each intervention studied. The score was calculated by assigning phase-based numeric value based on whether an intervention has a trial in a given phase and adding 0.001 for each trial in that phase to add significance for the existence of multiple trials in that phase. While the number of trials studying an intervention is important, an intervention with multiple Phase 1 or Phase 2 trials does not have the significance of a trial that has a successful Phase 3 trial and reaches regulatory approval as these interventions that do not progress are not readily applied in the real world. The scoring system accounts for this. For example, tocilizumab had 12 phase 3 trials so that would add 3.012 to the intervention score (3 for having a phase 3 trial and 0.12 [12 *0.001] for having 12 phase 3 trials). The higher the score the more significant the level of study of the intervention in the COVID-19 research landscape. For trials that combined two phases, we counted the trial as being of the higher phase (a phase 2/3 trial was considered just a phase 3 trial).

#### Publication attention score

Our goal was to generate a ranked list of publications with the most significant publications appearing on top. We used a construct of an attention score that gives the most significant publications higher values. The score is based on the recency of the publication, the phase of the trial, the intervention significance score, the number of times the trial record has been updated (high impact trials are more frequently updated), and whether the trial includes a US site. In other words, publications ranked higher if they were recent, from a later phase trial, involved a significant intervention, involved a CTG study record that had been updated multiple times and had at least one US site. For scoring purposes, if a trial was a combination of two phases, such as a phase 2/3 trial, we considered it under the higher phase (phase 3 in this example case).

We also retrieved article type from PubMed and gave publications that were not study result articles, such as protocols or editorials, less significance, and therefore lower attention scores, than study result articles.

In the final ranked publication list, we also present to the user further important publication and study metadata that are not input parameters for the calculation of the attention score. This information includes, the study sponsor, the journal where the publication was published, and whether study results were deposited on CTG as part of the trial record. This information can be seen in the supplemental material (regCovidpublications_Master.csv at the project repository).

#### Subset of COVID-19 vaccine trials

Due to the great importance and interest in vaccine trials for COVID-19, we looked specifically at a subset of COVID-19 vaccine interventional trials. The subset was developed by searching for the term vaccine in the trial’s title (developed and evaluated in the previously published regCOVID study; as of 2021, CTG does not capture vaccine as a separate intervention type) [4]. Similar to, the overall set of COVID-19 studies, we analyzed the vaccine trials based on key trial and publication features and generated attention scores for each publication associated with a trial of a COVID-19 vaccine.

#### Observational studies and registries

Along with the previously mentioned analysis of interventional trials, observational studies and registries were also analyzed. Similarly, to interventional trials, we identified both abstract and registry linked publications and assigned attention scores based on the recency of the publication, the number of study record updates and whether or not the study included a US site. Phase is not relevant for observational studies and registries.

## Results

All analytical results presented below were based on a query date of 31 January 2022. We plan to publish refreshed results at the study repository [12]. Repository history mechanism and formal data releases allow retrieval of any data release over time. The repository contains a report generated using an R notebook framework (computer code combined with user friendly result outputs). In addition to the report, important results are available as separate files in spreadsheet format. Such separate files are referred to in the results prefixed with ‘regCOVIDpublications_ ‘.

### Interventional trials

As of the query date (31 January 2022), a total of 3 167 recruiting, active or ended (completed or terminated) COVID-19 interventional trials (see file regCOVIDpublications_trials_int.csv) were identified and analyzed. On the trial level, a total of 565 trials (17.8% out of all 3 167 trials) have at least one linked result article. 197 (34.9%) trials have multiple publications, with 106 (18.8%) trials having published three or more articles.

The total number of trial-article-link-type combinations was 1 022, with 609 (59.6%) unique articles identified via abstract link and 413 (40.4%) identified via registry link. 27 (2.6%) articles overlapped and were identified via both link types. Since the same article can be linked to multiple trials (e.g., meta-analysis or an editorial about multiple trials), 926 distinct publications were linked to all included COVID-19 interventional trials.

It is important to consider the level of effort (of the principal investigator or other study officials) to link a publication to a trial. Abstract linking is easier and faster because the article author can simply state the NCT ID in the abstract and the article-study linkage is auto-generated thanks to the automated processing of PubMed abstracts. The majority of result articles (59.6%) were abstract-linked. On the other hand, registry linking requires update of the record in CTG by either XML file submission through their application protocol interface or by using CTG’s web-based data entry system (called Protocol Registration and Results System; PRS). Per our methodology, 1 170 registry-linked articles were removed as incorrect, misclassified result articles (articles that had a publication date prior to the start of the trial).

### Interventions

**Fig. 1:**
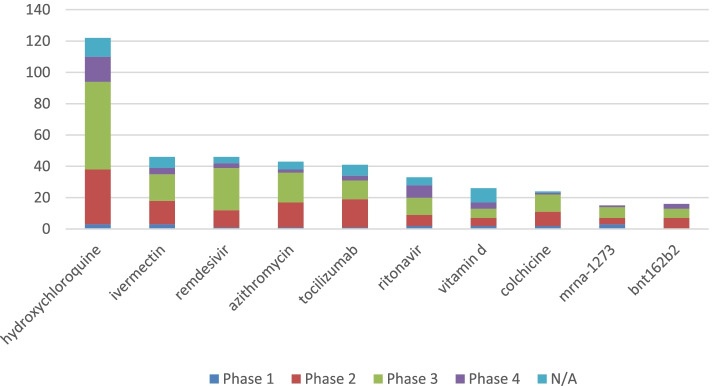
Count of trials by phase for select COVID-19 interventions

Using our computerized approach, we identified 4 036 interventions used in COVID-19 interventional trials. Of these 4 036 interventions, 784 had at least one publication connected to a trial. Table 1 shows a subset of interventions based on intervention score and includes the number of sponsors testing a given intervention, along with the number of publications resulting from these trials. Figure 1 shows the number of trials by phase for the same interventions. Data for all interventions (beyond those top 10 shown in Table 1) are available in file regCovid_intervention-phase_cnts_int2.csv as well as in the regCOVIDpublications report at the project repository.

**Table 1:** Counts of trials by phase, publications and sponsors aggregated by intervention

Intervention	Trial count	Intervention significance score	Number of sponsors	Number of publications
Hydroxychloroquine	122	11.122	102	86
Ivermectin	46	11.046	42	14
Remdesivir	46	11.046	31	28
Azithromycin	43	11.043	40	35
Tocilizumab	41	11.041	37	26
Ritonavir	33	11.033	25	25
Vitamin D	26	11.026	23	7
Colchicine	24	11.024	24	17
mrna-1273	15	10.015	7	12
bnt162b2	16	9.016	7	11

While Hydroxychloroquine was the intervention with the most publications (86) and highest intervention score (11.122) based on the number of trials and the breadth of the phases the trials covered, Convalescent Plasma was the intervention with the most distinct sponsors studying it (103). 850 interventions had at least one phase 3 (or phase 2/3) trial. While multiple vaccine candidates have progressed through each phase, the intervention significance score is lower than most other interventions that progressed to a similar phase since the volume of trials studying the vaccine candidate is usually limited by the fact that only the developer (and select co-sponsors) are studying the vaccine candidate. For example, the vaccine candidate mrna-1273 from Moderna has 15 total trials (three Phase 1, four Phase 2, seven Phase 3, and one Phase 4) with an intervention significance score of 10.015, which is lower than most other interventions that also proceed to phase 3 (as seen in Table 1) which have a much higher volume of total trials.

### Publication significance

Using the attention score to rank publications, we generated a ranked list of all 1 022 publications and a short list of 83 prioritized publications (publications that were not protocols, were from late phase trials (phase 3) with at least one US site and had multiple study record updates). Of the 1 022 trial-publication combinations, 309 (30.2%) were phase 3, 261 (25.5%) had at least one US site, and 742 (72.6%) had multiple study record updates.

Table 2 shows a subset of articles from the ranked list. For brevity, the table shows only a subset of available table columns. For the full list of 1 022 result article and trial combinations for COVID-19 interventional trials and the full spectrum of metadata (table columns), see supplemental file regCOVIDpublications_ publication_list_int.csv (master article list). The master article list aggregates metadata from both PubMed and CTG.

**Table 2:** A subset of publications with high attention scores

PMID	Article title	Publication date	NCT ID	Intervention^a^	Attention score
32,706,859	Remdesivir for Severe Coronavirus Disease 2019 (COVID-19) Versus a Cohort Receiving Standard of Care	12/13/2021	NCT04292899	Remdesivir|Standard of Care	5.116827
34,863,332	Lenzilumab in hospitalised patients with COVID-19 pneumonia (LIVE-AIR): a phase 3, randomised, placebo-controlled trial	12/11/2021	NCT04351152	Lenzilumab|Standard of Care	5.116714
33,972,949	LENZILUMAB EFFICACY AND SAFETY IN NEWLY HOSPITALIZED COVID-19 SUBJECTS: RESULTS FROM THE LIVE-AIR PHASE 3 RANDOMIZED DOUBLE-BLIND PLACEBO-CONTROLLED TRIAL	5/15/2021	NCT04351152	Lenzilumab|Standard of Care	5.104832
34,672,949	Efficacy of interferon beta-1a plus remdesivir compared with remdesivir alone in hospitalised adults with COVID-19: a double-bind, randomised, placebo-controlled, phase 3 trial	1/13/2022	NCT04492475	Interferon beta-1a|Placebo|Remdesivir	5.103581
34,407,339	Early Convalescent Plasma for High-Risk Outpatients with Covid-19	11/30/2021	NCT04355767	Convalescent Plasma|Saline	5.101792
33,204,764	Safety of Hydroxychloroquine Among Outpatient Clinical Trial Participants for COVID-19	6/22/2021	NCT04328467	Hydroxychloroquine|Placebo	5.099582
31,282,542	A Randomized, Placebo-Controlled, Pilot Clinical Trial of Dipyridamole to Decrease Human Immunodeficiency Virus-Associated Chronic Inflammation	2/5/2021	NCT04410328	Dipyridamole ER 200 mg/ Aspirin 25 mg orally/enterally AND Standard of care|Standard of care	5.09923

^a^ For presentation purposes, the following interventions are omitted in table (but present in full report): Placebo, Standard of Care and control

The use of the attention score and prioritizing certain facts about a trial and publication greatly reduces the list of all publications to a manageable list of publications for readers to review [83 publications compared to 1 022 publications]. Assuming a researcher may spend two minutes on each abstract, reviewing the full list versus the prioritized short-list results in a difference of 31.3 h in terms of total review time.

### Vaccine trial subset

For the subset of 436 total COVID-19 vaccine trials (as of the query date), at least one publication was for 64 (14.7%). For those 64 trials, there were 108 trial and publication combinations, with 92 (85.2%) being abstract linked. Due to the urgency and significant public interest in COVID-19 vaccines, we observed significant result articles published for trials that are formally ongoing, such as the Pfizer phase 2/3 trial for its vaccine candidates BNT162b1 and BNT162b2, which has already published six result articles, but does not have a listed completion date until 2 May 2023.

As of the query date, six vaccine trials have formally deposited basic summary results to the CTG registry, with one (NCT04498247) also having published a result article. Legal mandate allows for one year to do so for applicable US trials after the formal completion of the trial. This shows that vaccine trial sponsors may prefer publishing a result article in an academic journal as opposed to registry result deposition to communicate the results to the public. Although, this imbalance is also impacted by legal rules (for US-based trials) governing registry result deposition, namely, the official primary completion date and one year allowed legal time window after this date greatly influence when registry result deposition is performed.

### Observational studies and registries

Because of the mostly computerized nature of our analysis, the same analyses were executed on COVID-19 observational studies and registries. 661 result articles were found for observational studies. In contrast to interventional trials, more publications were registry linked (365 articles, 55.2%) than abstract linked (296 publications). On the study level, 339 COVID-19 observational studies (14.4% of all 2 350 COVID-19 registered observational studies) had at least one result publication.

175 result articles were found for registries and similarly to observational studies, the majority were registry linked (106 articles, 60.6% of the 175 publications). On a study level, 72 COVID-19 registries (20.1% of 359 total COVID-19 registered registries) had at least one linked study result article.

Unlike applicable interventional trials, US law does not mandate registration of observational studies or registries. A lack of a registration mandate does not allow for the determination of the proper denominator (to know the totality of COVID-19 observational studies or registries). The whole method of using abstract link or registry link (relying on NCT ID) naturally fails for unregistered studies. Researchers must rely on traditional PubMed searches to discover result articles of unregistered studies.

## Discussion

There are several prior analyses that report on how many studies provide results to the public. Huser et al. analysis from 2013 reported that 27.8% of analyzed interventional trials had published a linked result article [6]. A systematic review by Bashir et al. from 2017 found that a median of 23% (ranging from 13 to 42%) were linked to a result article [14]. With much increased public attention during the COVID-19 pandemic, we were motivated to find out what would be the percentage for COVID-19 studies. Our results, as of the query date, show that only 17.8% of COVID-19 interventional trials have a linked result article. However, it is too early to arrive at a formal number due to the relatively recent completion date (or formal ongoing status) of many trials.

Our methodology quickly identified result publications for prominent trials, such as trials involving vaccines approved in the US. Targeted review of those studies shows that such studies updated their CTG record frequently, which gives more confidence in the study metadata and study status (completed, terminated, or ongoing). In terms of paring trials with their result-reporting journal articles, the majority of linked result articles for interventional COVID-19 trials were found via abstract-link (59.6%), perhaps due to the easier practice of including the NCT ID in the article abstract.

The main advantage of our approach is offering researchers and the public a structured overview of literature with valuable metadata that combines information from scientific literature (PubMed) and clinical trial registry (CTG). It allows researchers to sort or aggregate articles based on various useful parameters (trial phase, sponsor, intervention and many others). Such capability is not possible with existing tools. Neither PubMed search nor clinical trial registry allow for review that would combine data from both sources. It allows for an overview of the clinical research in a given disease generated though automated computer script. For example, a review of all articles for a given intervention (such as hydroxychloroquine) could reveal if there is a consensus opinion on its efficacy or if there is a divide and more research is needed. In the case of hydroxychloroquine, a review of 12 results articles from six clinical trials in the US (on the prioritized short list) all expressed that the intervention was ineffective. A review of a full article master list (worldwide scope; not restricted to trials with at least 1 US site) would show a total of 97 articles from 40 trials studying hydroxychloroquine (see supplemental file for the master article list called ‘regCOVIDpublications_publication_list_int.csv’).

### Levels of trial visibility

Our results show various levels of trial result reporting ranging from zero to multiple result articles. 146 COVID-19 interventional trials were found that had multiple study result articles, as well as multiple registry record updates. On the next level are trials with exactly one result article. Considering trials with at least one linked journal article, 65.1% of those have exactly one article. Within the set of trials with exactly one article, 20.9% only had a publication of publication type protocol and not of publication type study result article, which is most valuable. Finally, the vast majority of COVID-19 trials do not have any linked result publications (2 602 studies, 82.2%), making it difficult for interested parties to know the outcome of the trial. An even more extreme case of minimal trial information are trials with no linked result articles and zero updates (besides the initial registration) to the CTG study record (506 interventional trials, 16.0% of 3 167 total interventional trials). Our project, regCOVID, is the first to utilize number of registry record updates (and the type of this update) as a novel, computed study metadata construct to further categorize studies by level of activity. This can be helpful in comparing studies with identical official study status and improve the prioritization of result publications stemming from these studies.

### Result deposition

As an alternative to publishing study results through an article, many studies chose to distribute study results by depositing them on CTG. A total of 146 trials deposited basic summary results. Within those, 56 trials only did registry result deposition and have no study result article, while the remaining 90 trials did both result deposition and published a result article.

### Trial registration timing

As part of our analysis, we found that trials register at three different points in time: [1] *prior* to trial initiation, [2] after trial initiation and prior to completion (*during*), and [3] *after* trial completion. A prior study showed that a majority of studies registered retrospectively, after the start of the study but prior to result publication [15]. Another study showed trial registration and timing may be affected by a variety of trial characteristics [16]. For the set of all COVID-19 trials the breakdown was 2 641 (44.9%) trials registered prior to trial initiation, 2 487 (42.3%) during the trial, and 748 (12.7%) after the trial completion. In comparison, when considering all studies initiated in 2020 (not restricted to COVID-19), 59.3% registered prior to starting, 27.1% registered during the study and 13.6% registered after the study was completed. The comparison shows that COVID-19 studies are more likely to register late (during the study; proportion of 42.3% for COVID-19 studies versus 27.1% for general studies).

### Publication timing

Publication of study results, including peer review, can be a complex and lengthy process. Prior studies indicate that it can take 21 months [17]. In a pandemic, like COVID-19, the quick publication of trial results is important for understanding which interventions are effective. Prior approval of COVID-19 vaccines and in the context of hospital staff and intensive bed shortages, clinicians were keen to learn about the efficacy of numerous tested interventions. A shorter publication timeline was targeted and seems to be apparent in prior studies [8]. Using our set of registered COVID-19 studies, on average, articles, that are not protocols, were published 214 days after the start of the trial. Trial start date was used as an anchor since many trials list on CTG anticipated completion dates in the future.

### Publishing prior to formal study completion

While primarily clinical trials publish study results articles after the formal trial completion date, for high profile trials it is not uncommon to see the opposite situation. During an ongoing pandemic, timely publication of results is important. For example, for the widely known trial regarding the Moderna COVID-19 vaccine (NCT04470427) which has an official primary completion date of 27 October 2022, the study result article was published in December 2020 (PMID: 33,378,609). This situation is, in fact, quite common. as 289 trial result articles linked to 130 COVID-19 trials are not formally completed as of the query date.

### Other considerations

#### Termination reason

The updating of the study registry record can be very important to the public and researchers. An especially important update is change of study status to terminated. Namely, the reason for termination can provide a highly valuable insight into the trial and studied intervention [18]. Such type of update is unlikely to be published as a separate article in a medical journal and the trial registry is the most suitable platform to communicate such an update. Of note is the fact that not all registries support record update and some may only focus on initial registration. To complement our intervention and publication prioritization, we also briefly analyzed the termination reason metadata supported by CTG registry. Most terminated trials (152, 87.4% of 174 terminated COVID-19 studies) specified a termination reason that helped explain why the trial was terminated. Most often, COVID-19 trials were terminated due to the inability to recruit and enroll participants. Other termination reasons were: intervention safety concerns, futility of the intervention, or availability of results from other trials making trial continuation unnecessary.

#### Publication bias

While manual review of abstracts of result publications was out of scope, we understand the potential presence of publication bias that may lead some trials to not formally publish results in a medical journal. For example, with reports of clearly terminated plans for further vaccine developments by some sponsors, a lack of result articles for certain trials and vaccine candidates hints at possible publication bias in vaccine trials.

#### Other manual trial trackers

Besides computational methods to obtain the most relevant COVID-19 journal articles, alternatively, it is possible to rely on websites (and research teams) that provide manually reviewed lists of completed studies with reported results. For example, The New York Times maintains a vaccine and therapy tracker [11]. Another study tracker is published by the NIH [19]. While it was out of scope to manually curate a sophisticated list of COVID-19 studies, or do a comprehensive review and comparison of our results to manual COVID-19 study trackers, we did compare the vaccine subset of COVID-19 studies identified through our methodology with those identified by the New York Times and NIH COVID-19 vaccine study trackers. Our motivation was to see how inclusive our methodology was. Our computerized approach study identification methods identified 29 of 37 phase 3 vaccine trials included in the New York Times vaccine tracker and included five of six trials present in the NIH study tracker.

### Generalization to other diseases: regCTGpublications

Due to the computerized nature of our methodology, the method and developed script can be applied to other conditions to achieve an analogous overview of interventions and ranked list of publications. Our project called regCTG [20] finds a list of studies for a given condition (generalization of regCOVID) [4]. A second project called regCTGpublications (or regCTGpub for short) generates a ranked list of result articles for trials in a given condition (generalization of regCOVIDpublications). The regCTGpub project repository [21] contains web-based result reports (analogous to Tables 1 and 2) for select medical conditions (such as Age-Related Macular Degeneration, Alzheimer, etc.).

### Limitations

Our study has several limitations. First, we rely on structured links between a registered study and the result article. A prior study for trials completed from 2004 to 2008 indicates that the negative predictive value of such a link may be as low as 56% [22]. In other words, an unlinked result article may exist for a trial. However, in recent years, journal requirements to include NCT trial identifiers in an abstract may now be better enforced. Second, researchers have no obligation to publish result articles in a medical journal. Our study uses indexed medical journal publications, though sponsors may make study results public via a press release, instead. Third, our study uses only a single, US-based, clinical trial registry: ClinicalTrials.gov, though, on the other hand, other registries often do not allow linking of a result publication in a registry record, don’t support basic summary result deposition and have limited or no API access options. Also, the CTG registry has a significant number of non-US studies: as of March 2021, 60% of studies in the recruiting status were non-US only. Fourth, one part of our algorithm, that can be turned off or re-configured for a different country, focused on trials with at least one US site. We chose this because some legal mandates are tied to this factor. Also, approval in the US (by Food and Drug Administration) is a significant factor in world-wide regulatory context (with some exceptions). Fifth, interventions are entered into CTG as free text and proper linkage of identical interventions (expressed using similar intervention strings, such as ‘anti-sars-cov-2 convalescent plasma’ and ‘convalescent covid 19 plasma’) depends on a computational algorithm that can miss some linkage of identical interventions.

## Conclusion

We developed a data science driven approach to quickly identify and track linked articles for COVID-19 clinical studies and characterized which studies are publishing, what type of trial-article link is used, and designed a ranking score to prioritize the most significant publications for understanding clinical research for COVID-19. For a set of 3 167 active or ended interventional trials, 1 022 published study result articles were identifed, including a prioritized list of 83 key articles from late phase, US based trials with multiple study updates. We separately analyzed trials for COVID-19 vaccines and found 108 linked result articles (including the Pfizer/BioNTech, Moderna and Johnson and Johnson vaccine trials). Our approach gives researchers and health care professionals a quick overview of the state of COVID-19 clinical research and allows for the most efficient review of clinical study results. The computerized nature of our research also allows for consistent and as needed updating of results and is easily generalizable to any condition of interest.

## Data Availability

The datasets generated and/or analyzed during the current study are available in the regCOVID and regCOVIDpublications repositories, r-snippets-bmi/regCOVID at master lhncbc/r-snippets-bmi GitHub and r-snippets-bmi/regCOVID/regCOVIDpublications at master lhncbc/r-snippets-bmi GitHub.
